# Influence of unsaturated to saturated ratio of fatty acids reaching the duodenum on postruminal digestion of stearic acid in Holstein steers fed a high-fat finishing diet

**DOI:** 10.5455/javar.2021.h535

**Published:** 2021-09-20

**Authors:** Alejandro Plascencia, Alberto Barreras, Yissel Valdés-García, Richard A. Zinn

**Affiliations:** 1 Departamento de Ciencias Exactas y Naturales, Universidad Autónoma de Occidente, Guasave, México; 2 Instituto de Investigaciones en Ciencias Veterinarias, Universidad Autónoma de Baja California, Mexicali, México; 3 Department of Animal Science, University of California, Davis, CA, USA

**Keywords:** Fatty acid, postruminal digestion, steers, stearic acid, oleic acid, finishing diets

## Abstract

**Objective:**

To evaluate the influence of the unsaturated to saturated ratio of fatty acids (FAs) reaching the duodenum on postruminal digestion of FAs, mainly focused on stearic acid (C18:0).

**Materials and Methods:**

Six Holstein steers [208 ± 3 kg initial live weight (LW)] with cannulas in the abomasum and proximal duodenum were used in a replicated 3 × 3 Latin square design. Steers were fed a fixed amount of a basal steam-flaked corn-based diet containing 8% supplemental fat and were daily infused via abomasum with 0, 67, and 165 gm oleic acid (C18:1). The experiment lasted for 42 days.

**Results:**

The daily total FA (TFA) intake (dietary FA intake plus abomasal infusion of oleic acid) represented a 1.78, 2.10, and 2.56 gm TFA/kg LW ratio. The unsaturated to saturated ratio of FAs entering the duodenum increased (*p* < 0.01) as level C18:1 infusion into the abomasum increased. Infusion of C18:1 tended (quadratic component, *p* = 0.07) to improve postruminal TFA digestion, being maximal for the 67 gm/day infusions. This increase in TFA digestion was due to increased (quadratic component, *p* = 0.03) postruminal C18:0 digestion (postruminal digestion of the other FAs was not different, *p* ≥ 0.13).

**Conclusion:**

Increasing the unsaturated to saturated ratio of FAs entering the small intestine will enhance intestinal C18:0 digestion. This positive effect is expected to be more likely beneficial when FA intake is high (and thus, the duodenal flow of FA is high), but this benefit looks diminished when the quantity of TFA reaching the intestine exceeds the proportion of 2.13 gm FA/kg LW.

## Introduction

The net energy of maintenance (NE_m_) value assigned by the current standards [1] for supplemental fats for feedlot cattle is from 5.65 to 6.03 Mcal/kg. In as much as feed-grade fats largely (~90%) comprise fatty acids (FAs), their net energy value is closely associated with FA digestibility [2]. Fat intake level has the greatest impact on fat digestion [3,4]. This effect results from the limitations on intestinal absorption of saturated FA due to upper limits on bile production [5]. Another factor that limits intestinal FA digestion is the saturated-to-unsaturated FA ratio reaching the intestine. As a consequence of ruminal biohydrogenation of unsaturated FA, C18:0 accounts for 65% or more of the total FA (TFA) entering the intestine. Stearic acid is absorbed almost similarly (90%) to that of unsaturated FA when fat is supplemented in diets at lower levels (i.e., <3%), but this proportions can drop to 80% or less at higher (~6%) levels of fat supplementation [6]. Intestinal digestion of C18:0 alone explains >85% of the variation in net energy value of supplemental fat [7]. As a result, the energy value of supplementary lipids can be increased by lowering ruminal biohydrogenation and/or improving intestinal digestibility of C18:0. On the latter, a synergistic effect of small intestine absorption of saturated FAs in broilers has been determined when the proportion of the unsaturated to saturated ratio that reaches the intestine is raised [8], increasing the energy value of supplemental fats. Likewise, in ruminants, protecting supplemental fat from ruminal biohydrogenation improves intestinal digestion of C18:0. Zinn et al. [9] reported that for each percentage unit increase in the proportion of unsaturated FA (mainly oleic acid) entering the small intestine, digestibility of C18:0 increased by 1%. This could be explained by oleic acid’s significant amphiphilic properties, which increase the surface area of the bile salt micelle, increasing its hydrophobic capacity and thus alleviating the potential limitation of bile production associated with high-fat intakes, thereby improving the physical–chemical conditions for increased intestinal absorption of saturated FAs [4].

Due to the intense ruminal biohydrogenation of unsaturated FAs, it is complex to precisely manipulate the proportions of saturated to unsaturated FAs that reach the intestine. In ruminants, the reports on this subject are minimal. To our knowledge, the effect of an increasing proportion of unsaturated to saturated FAs flowing to the duodenum on the postruminal digestion of saturated FAs in cattle fed high-fat finishing diets has not been directly assessed. The purpose of this experiment was to determine the effect of increasing the proportion of unsaturated to saturated FAs in Holstein steers fed a high-fat finishing diet on postruminal digestion of stearic acid (C18:0). This was accomplished by directly infusing oleic acid (C18:1) into the abomasum (thereby avoiding ruminal biohydrogenation of oleic acid).

## Material and methods

### Ethical approval

The protocol of this experiment was approved by the University of California Animal Care and Use Committee (protocol #18811).

### Characteristics of the experimental units, basal diet, treatments, and sampling methods

Six Holstein steers [208 ± 3 kg live weight (LW) at start of the experiment] with cannulas in abomasum [10] and proximal duodenum [tygon “T” cannula (1.9 cm i.d.) placed approximately 6 cm from pyloric sphincter] were used in a replicated 3 × 3 Latin square experiment to evaluate the influence of abomasal oleic acid infusion (C18:1) on characteristics of postruminal FA digestion. The steers were maintained in an individual neoprene-floor pen (2.35 × 3.92 m) and drawer feeder with *ad libitum* access to water. Intake of basal diet was limited to 4.60 kg/day (as is fed basis), which was equivalent to 2.2% of the initial LW or 74.6 gm of diet dry matter (DM)/kg LW^0.75^), offered at equally twice daily (0800 and 2000 h) during all experiments which lasted for 42 days. Ingredient composition and chemical characteristics of the basal diet are shown in Table 1. The external marker chromic oxide (Cr_2_O_3_) (4.0 gm/kg of diet) was employed to assess digestion coefficients. Before incorporating into the diet, the external marker was premixed for 5 min with other minor ingredients (trace mineral salt, limestone, and urea) using a 2.5 m capacity mixer. The treatments were as follows: 1) purified water infused via the abomasal cannula at 2.79 ml/h (67 gm/day); 2) oleic acid-infused via the abomasal cannula at 3.14 ml/h (67 gm/day), and 3) oleic acid-infused via the abomasum cannula at 7.72 ml/h (165 gm/day). The oleic acid-infused was oleic acid NLT 98% (relative density = 0.89; Cas# 112-80-1 Mallinckrodt Baker Inc., Phillipsburg, NJ). The infusion was accomplished using a variable flow peristaltic pump (Variable Flow Mini-Pump II, VWR Scientific Products, West Chester, PA). Infusion tubing (3.1 mm d.i. × 5.1 m Tygon^®^; Norton, Akron OH 44309-3660) was moored at the ceiling above each pen using 1 m lengths of 6.3 mm latex tubing to provide constant tension, thus permitting free movement of steers during infusion. The experiment lasted for 42 days and consisted of 3 experimental periods of 14 days planned as follows: 3 days for infusion treatment adjustment, followed by a sampling period of 4 days; then, following the completion of each infusion period, steers were rested (free of infusion tubing) for 7 days but the intake of basal diet was maintained. Duodenal and fecal samples were taken from each steer following the procedure (sample quantity and schedule sampling) described by Núñez-Benítez et al. [11]. Once the sampling period finished, duodenal and fecal samples from each steer and within each sampling period were composited for subsequent analysis.

**Table 1.** Composition of the basal diet fed to cannulated Holstein steers.

Ingredient composition	% in diet
DM content	88.82
Ingredient composition	DM basis
Alfalfa hay	6.00
Sudangrass hay	6.00
Steam flaked corn	55.85
Steam flaked wheat	10.00
Tallow	8.00
Cassava	7.00
Cane molasses	3.00
Limestone	1.37
Urea	1.07
Chromic oxide	0.40
Dicalcium phosphate	0.16
NaHCO_3_	0.75
Trace mineral salt^a^	0.40
Nutrient composition, % DM basis^b^	
Calculated crude protein	11.92
Calculated neutral detergent fiber	13.66
Determined TFA	9.05
Calculated NE, Mcal/kg	
Maintenance	2.38
Gain	1.68

a Trace mineral salt contained: CoSO_4_, 6.8 gm/kg; CuSO_4_, 10.4 gm/kg; FeSO_4_, 35.7 gm/kg; ZnO, 12.4 gm/kg; MnSO_4_, 10.7 gm/kg; KI, 0.52 gm/kg; and NaCl, 923.5 gm/kg.

b Calculated from the tabular values for individual feed ingredients [1]; average DM and FA concentration of basal diet were determined in subsamples gathered and composited throughout the experiment.

### Laboratory analysis

Feed, duodenal, and fecal samples were subjected to the DM (oven drying at 105°C until no further weight loss) determination, Cr_2_O_3_ determination [12], and FA determination. According to Zinn and Plascencia’s [3] protocols, direct methyl esterification was used to identify FAs [13], followed by gas chromatographic analysis [14]. The Cr intake estimated the total DM flow to the duodenum and excreted in feces (gm/day) versus concentration of Cr in the duodenal and fecal samples as follows: total DM output, gm/day = gm Cr_2_O_3_ intake daily/(g Cr_2_O_3_/gm of duodenal/feces). 

### Statistical analysis

The effects of the treatments on the characteristics of postruminal FA digestion were analyzed as a replicated 3 × 3 Latin square design using the MIXED procedure of the Statistical Analytical System software [15]. The fixed effects consisted of treatment and period, and steer as a random effect. The statistical model for the trial was as follows: *Y*_*ijkl*_ = *μ* + *C*_*i*_ + *A*_*j*_ + *P*_*k*_ + *E*_*ijk*_, where *Y*_*ijk*_ is the response variable, *C*_*i*_ is the oleic acid level effect, *A*_*j*_ is the animal effect, *P*_*k*_ is the period effect, and *E*_*ijk*_ is the residual error. Orthogonal polynomials were used to investigate the linear and quadratic effects of oleic acid infusion. Contrast coefficients for u*nequally spaced* levels of oleic acid infusion were carried out using the ORPOL function [15]. Treatment effects were considered significant when *p*-value was ≤0.05, and tendencies were identified when *p*-value was >0.05 and ≤0.10. 

## Results and Discussion

In as much as DM intake of the basal diet was constant for all steers, differences in FA intake and saturated to unsaturated FA ratio between treatments was due to the C18:1 infused into the abomasum (Table 2). Accordingly, the daily relative FA intake (expressed as gm FA/kg LW) linearly increased (*p* < 0.01) from 1.78 (0 gm infused) to 2.63 (165 gm infused/day), while the proportion of unsaturated to saturated FA intake linearly increased (*p* < 0.01) from 1.10 (approximately 52% unsaturated FA) to 2.02 (approximately 67% unsaturated FA). 

Duodenal flow, fecal excretion, and postruminal digestion of FA are shown in Table 3. Considering all treatments, the average TFA flow to the small intestine was 12% greater (average = 498 gm/day) than the average FA intake (446 gm/day). This differential reflects the ruminal indigestibility of dietary FA in combination with ruminal microbial FA synthesis. Analyzing data from other experiments carried out with feedlot cattle fed high-energy diets supplemented with fats which report FA intakes and FA intestinal flows [5,9,16–19], the relationship between FA intake versus the duodenal FA flow is as follows: *Y* = 1.134*X* (*R*^2^ = 0.91), where *Y* = duodenal FA flow (gm/day) and *X* = FA intake, gm/day. When the equation is applied to the current experiment, the average anticipated flow is 505 gm/day, which is quite near the observed value (0.99). When no C18:1 was infused, the saturated to unsaturated ratio of FAs reaching the duodenum was 4.98. Duodenal flow (as % of TFA flow) of saturated FAs increased 1.7-fold (82.0%) when compared with the proportion of 47.5% of saturated FAs in the TFA intake. This effect is due to ruminal biohydrogenation of unsaturated FAs [20], which typically average 70% (ranging between 60% and 90%) [7]. As expected, the saturated to unsaturated ratio of FA entering the duodenum declined (*p* < 0.01) as level C18:1 infusion into the abomasum increased.

Infusion of C18:1 tended (quadratic component, *p* = 0.07) to increase postruminal FA digestion, being maximal for the 67 gm/day infusions. This increase in TFA digestion was due to increased (quadratic component, *p* = 0.03) postruminal C18:0 digestion (postruminal digestion of the others FA was not different, *p* > 0.13). Due to its greater facility for the formation of micelles, small intestine absorption of unsaturated FAs is greater than the saturated FAs [7]. Thereby, in marked difference with saturated, the intestinal digestion of unsaturated FA is hardly affected when their supply to the small intestine increases [21]. Accordingly, intestinal FA digestion and hence, the feeding value of fat is a function of FA intake level and the expected profile of FAs entering the small intestine. 

**Table 2.** Ingestion of FA in steers (208 kg LW) infused with 0, 67, or 165 gm of oleic acid/day.

Item	C18:1 infusion, gm/day		
	0	67	165
Intake, gm/day			
DM^a^	4,086	4,154	4,222
FAs (from feed)	370	376	382
FAs infused	0	67	165
TFA intake	370	443	547
FA intake, gm FA/kg LW	1.78	2.13	2.63
Saturated FA intake	176	176	176
Unsaturated FA intake	194	260	356
Unsaturated: saturated FA ratio	1.10	1.48	2.02

a Include DM of feed plus DM of FA infusion.

**Table 3.** Treatments’ effects on postruminal digestion of FA of Holstein steers (208 kg LW).

Item	C18:1 infusion, gm/day			SEM	Contrast *p*-value	
	0	67	165		*L*	*Q*
FA intake	370	443	547	--	--	--
Flow to duodenum, gm/day						
TFA	400	478	595	28.9	0.04	0.99
C14:0	16.5	16.6	16.2	0.12	0.31	0.35
C16:0	124	143	145	12.3	0.39	0.55
C18:0	192	192	225	9.96	0.13	0.39
C18:1	49	103	180	5.08	<0.01	0.80
C18:2	18.5	23.3	29.3	2.31	0.08	0.90
Saturated FAs	333	352	387	22.3	0.22	0.94
Unsaturated FAs	68	126	209	6.30	<0.01	0.80
Unsaturated: saturated FA ratio	0.20	0.36	0.54	0.03	<0.01	0.03
Fecal excretion, gm/day						
TFA	108	88	173	19.7	0.12	0.19
C14:0	1.42	3.46	2.52	0.89	0.55	0.28
C16:0	30.0	26.9	41.8	15.2	0.61	0.72
C18:0	70.3	43.9	112.6	4.0	0.01	<0.01
C18:1	4.87	10.9	14.4	4.45	0.28	0.72
C18:2	1.35	1.89	1.95	0.43	0.45	0.63
Saturated FAs	102	74	157	15.64	0.10	0.12
Unsaturated FAs	6.23	12.8	16.3	4.66	0.27	0.70
Unsaturated: saturated FA ratio	0.06	0.17	0.10	0.07	0.14	0.27
Posruminal digestion, %						
TFA	73.02	81.58	70.92	2.24	0.52	0.07
C14:0	91.40	78.76	84.52	5.91	0.56	0.30
C16:0	75.63	82.25	73.25	9.36	0.82	0.58
C18:0	63.22	77.05	49.51	2.71	0.06	0.03
C18:1	89.95	89.97	92.21	3.50	0.68	0.86
C18:2	92.64	91.21	93.11	1.61	0.80	0.50
Saturated FAs	69.31	78.96	59.81	2.33	0.08	0.04
Unsaturated FAs	90.83	89.84	92.30	3.15	0.72	0.77

With regard to finishing diets commonly offered for feedlot cattle in the USA, Plascencia et al. [6] informed that accurately known TFAs intake and intestinal digestion of FAs can be predicted as follows: FA digestion (gm/kg LW): *Y* = 87.560 − 8.591*X* (*R*^2^ = 0.89, *n* = 25), where *Y* = intestinal digestion and *X* = FA intake per day (gm/kg LW). Due to observed limitations of FA intake on subsequent FA digestion, the authors concluded that finishing diets should be formulated not to exceed 0.88 g FA/kg LW (approximately 0.98 gm lipid/kg LW). According to their equation, the estimated postruminal FA digestion rates are 72.3%, 69.3%, and 65.8% for infusions of 0, 67, and 165 gm/day, respectively. The observed postruminal FA digestion for 0 infused FA treatment was in close agreement (101%) with the expectation. However, for the 67 and 165 gm/day FA infusion treatments, the observed postruminal FA digestion was 15.1% and 7.2% greater than expected, respectively. 

A synergistic effect of small intestine absorption of saturated FAs in broilers when the proportion of unsaturated to saturated FAs ratio that reaches the intestine is raised has been reported [8]. In a similar manner, reducing the ruminal biohydrogenation index of the unsaturated FAs by protecting fat of the ruminal biohydrogenation increases TFAs absorption from 80.3% to 87.8% [9]. It has been established that there is a direct relationship (1:1) between the increase in the proportion of unsaturated to saturated FAs that reach the intestine and the increase in C18:0 intestinal digestibility. Nevertheless, limitations on intestinal FA digestion are also constrained by limits in bile production. Plascencia et al. [5] observed that bile production in cattle is relatively constant, and unlike non-ruminants, refractory to the level of fat intake, explaining 69% of the variation in postruminal FA digestion. In such a way that even when high proportions of unsaturated FAs reach the intestine, the potential for bile production will be the limiting factor for its absorption.

Based on the physiological fuel value of lipids (9 kcal metabolizable energy per gram) and the efficiency with which this energy can be used for weight gain [7], the net energy value for gain (NE_g_) of dietary fat is 6.03 kcal/gm of intestinally absorbable fat. Therefore, NE_m_ and NE_g_ values for supplemental tallow were 5.52 and 4.40, 6.16 and 4.95, and 5.40 and 4.33 for 0, 67, and 165 gm/day infusions, respectively. These findings demonstrate that increasing the fraction of unsaturated FA reaching the small intestine improves FA digestion in ruminants and non-ruminants alike. The extent of this increase in FA usage on growth performance responses, on the other hand, is uncertain. Additional research is needed to determine the effects of this method on feedlot cattle performance.

## Conclusion

Decreasing saturated to unsaturated ratio of FAs entering the small intestine will enhance intestinal C18:0 digestion. Accordingly, protecting supplemental fat from ruminal biohydrogenation is an alternative to improve intestinal FA digestion and hence, the energy value of dietary fat in finishing diets for feedlot cattle. However, TFA supply to the small intestine is a major constraint on intestinal FA digestion (by limited bile production). Modifications to the unsaturated to saturated ratio of FAs entering the small intestine is expected to be more likely beneficial when expected FA flow to the intestine does not exceed 2.13 gm FA/kg LW.

## List of Abbreviations

Cr_2_O_3_: chromic oxide, DM: dry matter, gm: gram, kg: kilogram, LW: live weight, NE_g_: net energy of gain, NE_m_: net energy of maintenance, NLT: not less than, ORPOL: orthogonal polynomials, SAS: Statistical Analytical System, TFA: total fatty acids.
